# Different Learning Curves for Axillary Brachial Plexus Block: Ultrasound Guidance versus Nerve Stimulation

**DOI:** 10.1155/2010/309462

**Published:** 2011-01-20

**Authors:** C. Luyet, G. Schüpfer, M. Wipfli, R. Greif, M. Luginbühl, U. Eichenberger

**Affiliations:** ^1^Department of Anesthesiology and Pain Therapy, University Hospital and University of Bern, Inselspital, CH-3010 Bern, Switzerland; ^2^Department of Anesthesiology, Intensive Care, Emergency Medicine and Pain Therapy, Kantonsspital Lucerne, 6000 Lucerne, Switzerland

## Abstract

Little is known about the learning of the skills needed to perform ultrasound- or nerve stimulator-guided peripheral nerve blocks. The aim of this study was to compare the learning curves of residents trained in ultrasound guidance versus residents trained in nerve stimulation for axillary brachial plexus block. Ten residents with no previous experience with using ultrasound received ultrasound training and another ten residents with no previous experience with using nerve stimulation received nerve stimulation training. The novices' learning curves were generated by retrospective data analysis out of our electronic anaesthesia database. Individual success rates were pooled, and the institutional learning curve was calculated using a bootstrapping technique in combination with a Monte Carlo simulation procedure. The skills required to perform successful ultrasound-guided axillary brachial plexus block can be learnt faster and lead to a higher final success rate compared to nerve stimulator-guided axillary brachial plexus block.

## 1. Introduction

Ultrasound-guided regional anaesthesia, requires the mastering of different skills: knowledge of physics, use of the ultrasound machine, improved manual dexterity, and extensive knowledge of sonographic anatomy are all needed. On the other hand the use of a nerve stimulator to detect vicinity of the needle to a nerve also requires knowledge of physics as well as knowledge of physiology and pathophysiology. The correct use of a nerve stimulator also deserves an adequate teaching. The acquisition of all these different skills can be especially challenging for the novice.

Learning curves comparing different manual anesthesia techniques provide figures that demonstrate the minimum number of cases required for each procedure to achieve a high success rate and define the competence level [1–4]. Little is known about the process of learning the skills required to perform ultrasound-guided blocks, for example, the number of blocks needed to acquire proficiency. Till now, no study has compared the success rates of ultrasound-guided nerve blocks with nerve stimulation during the learning process. 

The aim of this study was to generate novices' learning curves for nerve stimulator-guided axillary brachial plexus block by retrospective data analysis of the resident's anesthesia records before the ultrasound was introduced and to compare them with the generated learning curves for real-time ultrasound-guided axillary brachial plexus block after introducing the ultrasound technique at our department. The goal of this study was primarily to demonstrate a possible difference between the learning curves for each technique and to compare any specific side effects or complications (vascular puncture). The null hypothesis stated learning curves for either technique will not differ from one another significantly after the first 20 attempts.

## 2. Methods

The study was performed at the Department of Anesthesiology and Pain Therapy at the Bern University Hospital after general approval of the ethics committee for retrospective data analysis.

Before June 2006, the multistimulation technique was standard practice for axillary brachial plexus block in our department. We applied the multistimulation technique as described by Sia et al. [5, 6] starting with the median, followed by radial or ulnar nerve and finally musculocutaneous nerve, eliciting a distal motor response for each nerve. To achieve sufficient needle-nerve proximity, the motor response had to be present at a decreasing current intensity between 0.3 to 0.5 mA with preset pulse duration of 100 ms and a frequency of 2 Hz. Instruction of the nerve stimulation technique was performed individually and included technical instruction on the use of the nerve stimulator, anatomy teaching using a regional anaesthesia manual (Meier and Büttner [7]), and demonstration on one patient. The first ten blocks were performed under direct supervision by the staff anesthesiologist, thereafter the residents continued independently but with a staff anesthesiologist present in the operating room and on call for help at anytime. 

From September 2006, a few months training in ultrasound-guided regional anesthesia for the staff was provided before ultrasound was broadly introduced and taught in the clinical practice. Training for the staff consisted of a 2-day workshop in a specialized clinic in Vienna as well as training under supervision by an in-house expert in ultrasound-guided procedures. After this period, the same staffs were responsible for instruction of the ultrasound technique to the residents. Two lectures including basic principles of ultrasound, the use of the ultrasound device and the specific ultrasound anatomy were given to all residents. Two afternoons of practical workshops training in scanning and in needling techniques using both phantoms and models were performed prior to patient contact. Moreover, there was open access to phantoms/chicken drumsticks for all residents. After one demonstration in the operating room each resident performed 10 blocks under the direct supervision of a staff anesthesiologist, thereafter, the residents performed independently with a staff anesthesiologist present in the operating room on call for help at anytime. For real-time ultrasound-guided technique we used a high-definition ultrasound device (MicroMaxx, SonoSite Inc, Bothell, WA 98021-3904) with a 5–10 MHz linear array transducer (L38e, 10–5 MHz, 38-mm broadband linear array, SonoSite Inc, Bothell, WA 98021-3904). Ultrasound-guided axillary brachial plexus block was routinely performed with a 22 G insulated needle (Polymedic UPC 50, TeMeNa SAS, F-Charrières-sur-Seine) connected to a nerve stimulator (Stimuplex HNS 11 B. Braun Medical, D-Melsungen) with a fixed stimulation output of 0.3 mA (0.1 ms impulse width). The target nerves were identified by ultrasound and the needle tip was advanced under direct visualization close to the nerve. Needle guidance was performed using an out-of-plane technique. The local anesthetic was then injected under direct visualization around the targeted nerves. The nerve stimulator was used for two purposes: (1) in case the needle tip was lost on the ultrasound screen a motor response would indicate nerve contact. In such a case, the injection of local anesthetics was omitted in order to avoid an intraneural injection. (2) To confirm nerve identity when a motor response was present.

Although the introduction to the ultrasound technique requires more information to be given, that is, about the ultrasound device itself or how to perform an ultrasound exam, and so forth. practical training in the operating room was comparable. The same three staff anesthesiologists were responsible for instruction of the residents before and after introduction of the ultrasound technique.

All data were collected by retrospective analysis of the anesthesia electronic database after obtaining institutional ethics review board approval as well as written informed consent from the residents. The electronic database was created in 2000. Each handwritten intraoperative anaesthesia record, as well as the preoperative and postoperative records were scanned into the electronic database. Beside all drugs given during anesthesia, details of the block procedures were recorded. The occurrence of paresthesias, inadvertent vascular puncture, and local anesthetics given by the surgeon in case of a required block supplementation were meticulously documented. Because the effect of the block was not recorded in a standardized way, block success was defined according to clinical efficacy (see below). Other outcome measures like onset, intensity, or extent of the block were not recorded.

Anesthesia records from residents who started the training in one of these two methods at our department between 2000 and 2008 were analyzed. All residents in the nerve stimulation group had no previous experience in performing peripheral nerve blocks at all. Only four residents starting ultrasound-guided peripheral nerve block were already familiar with the nerve stimulation technique (having performed more than 20 nerve stimulator-guided axillary brachial plexus blocks). No residents in the ultrasound group had any previous experiences in performing ultrasound-guided peripheral nerve blocks. Their learning curves were analysed separately (the mixed group). All axillary brachial plexus block records were sorted chronologically. The records of the nerve stimulation group dated from February 2000 to December 2004, the records of the ultrasound group dated from September 2006 to March 2008. The time between 2005 and 2006 was excluded from analysis in order to avoid any bias due to the growing knowledge of ultrasound-guided regional techniques and any subsequent contamination of teaching.

From the records retrieved from the database, a chronological binary table of successful or failed axillary brachial plexus blocks for each resident was created. A failure was defined as block supplementation by the surgeon, need for deep sedation with propofol, ketamine, or conversion to general anesthesia. Furthermore, the following notes on the anesthesia records were accounted as block failure: if a part of the block was performed by the staff member, if the staff manually intervened to complete the block, or performed a rescue block before surgery.

The number of inadvertent vascular punctures, the time to perform the block, and the type and amount of local anaesthetics documented by the present anesthesia nurse were all recorded as secondary endpoints. 

### 2.1. Data Analysis and Statistics

No sample size calculation was performed due to a lack of analysis methods for comparing learning curves. The first 10 residents who were trained in ultrasound-guided puncture were systematically analysed. For the time between 2000 and 2004, we randomly picked out ten residents who were known to be novices for nerve stimulator-guided peripheral nerve block. 

From the binary data, individual success rates were computed, pooled among all participants, and compared between the groups of residents. The institutional learning curve was calculated by applying a fitting model with a Monte Carlo procedure, a random number simulation technique to mimic a statistical population [1, 4]. To calculate the 95% confidence intervals, the data were boot strapped [8]. The confidence intervals were used to create the institutional learning curves [4]. We defined the highest point of the success rate during the learning phase as “levelling-off.” This point gives an approximation of the number of procedures required to achieve the final success rate—or in other words, the “number needed to learn.” The differences in success rates of the nonextrapolated data were compared using a chi-square test with Bonferroni adjustment, therefore a *P*-value smaller than  .01 was considered statistically significant. Comparison of the preoperative patient's characteristics and the axillary brachial plexus block characteristics were made either by using Student's *t*-test or Mann Whitney rank sum test. Proportions were analysed with chi-square test. A probability of less than  .05 was considered significant. All calculations were performed by using SigmaStat for Windows Version 3.5.

## 3. Results

A total of 602 anesthesia records of ten residents in the nerve stimulation group and ten residents in the ultrasound group were reviewed. In the ultrasound group, there were four residents already familiar with the nerve stimulation method so this group was further divided in a mixed subgroup for constructing the learning curves. The nerve stimulation group counted 343 anesthesia records compared to 259 anesthesia records for the ultrasound groups (127 records for mixed). The groups were similar with regards to preoperative patient characteristics and surgical procedures (Table 1).

**Figure 1:**
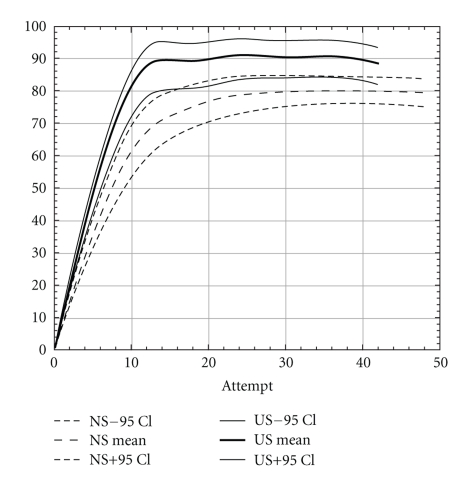
Institutional learning curves of the ultrasound group compared to the nerve stimulation group. The different endpoints of the curves reflect that more blocks beyond 40 attempts were evaluated in the nerve stimulation group.

**Figure 2:**
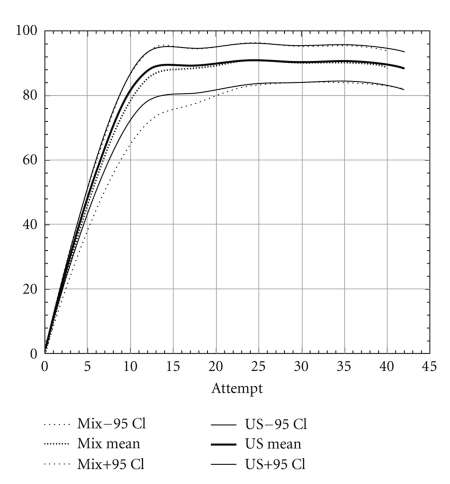
Institutional learning curves of the ultrasound group compared to the mixed group (novices for ultrasound but already experienced with the nerve stimulator). The confidence intervals for the two learning curves are mostly overlapping.

**Figure 3:**
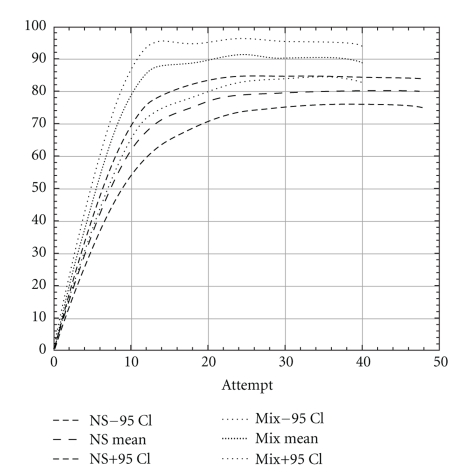
Institutional learning curve of the mixed group compared to the nerve stimulation group. Note: the different endpoints of the curves reflect that more blocks beyond 40 attempts were evaluated in the nerve stimulation groups.

Overall success rates for ultrasound-guided blocks (both groups) after 40 blocks was 89% (95% CI 85–93) which is significantly higher than the success rate of 80% (95% CI 75–84) in the nerve stimulation group after 40 blocks (*P* = .002). The difference was also significant after the first 10, 20, or 30 blocks (Table 3 shows results from the un-extrapolated raw data). When comparing the learning curves (extrapolated data), for ultrasound, the number needed to learn was between 10 and 15 whereas for nerve stimulation it was between 25 and 30 attempts (Figure 1). The learning success for ultrasound-guided axillary brachial plexus block of residents already familiar with the nerve stimulator (mixed group) was slightly lower. The learning curve of this group was found to lay between the nerve stimulation and ultrasound groups without significant difference between them (Figures 2 and 3). For ultrasound-guided blocks, residents used a smaller volume of local anesthetics compared to the volume of local anesthetics used for nerve stimulator-guided blocks (38 ± 6.3 mL versus 46 ± 6.8 mL; *P* < .001). The type of local anesthetics used was also slightly different. In the nerve stimulation group, there were more combinations of long-acting (Bupivacaine 0.5%) with short-acting local anesthetics (Mepivacaine 1%), whereas in the ultrasound group more blocks were performed using solely long-acting agents. The reasons for these differences were as follows (1) Between 2004 and 2006 there was a change in our department from the use of bupivacaine 0.5% to ropivacaine 0.75% as standard long-acting local anesthetic. (2) Ropivacaine was not mixed with mepivacaine as this was done with bupivacaine in the years prior. Another difference was the block-performing time, which was lower for ultrasound-guided blocks compared to nerve stimulator-guided blocks (22 ± 8 Minutes versus 35 ± 13 Minutes; *P* < .001). Of all 343 nerve stimulator-guided blocks there were 173 vascular punctures (50%; 95% CI 45–56). Of the 259 blocks of the ultrasound group there were 32 vascular punctures (12% 95% CI 9–17). This difference was highly significant (*P* < .0001). The absolute risk reduction of inadvertent vascular puncture is 38% (95% CI 31–44) (Table 2).

## 4. Discussion

The popularity of real-time ultrasound guidance for nerve blockade has increased dramatically over the last 10 years. A few studies have shown that the use of ultrasound improves the success rate of axillary brachial plexus block when compared with nerve stimulation [9, 10] or with the transarterial technique [10, 11]. An alternative study by Casati et al. [12] however, could not demonstrate this improved success rate. Benefits of the use of ultrasound are the reduced need for nerve stimulation with improved patient comfort [12], reduced volume of local anesthetics used [13–16], and shortened onset time [10, 17]. In this retrospective study focussing on learning of the skills, we found that ultrasound-guided axillary brachial plexus block when performed by junior residents is learned faster and with a higher success rate compared to nerve stimulator-guided axillary brachial plexus blocks. Furthermore, there were significantly less vascular punctures when using ultrasound.

The learning curves for ultrasound-guided axillary brachial plexus blocks showed a stronger upsurge compared to nerve stimulator-guided axillary brachial plexus blocks and the levelling of the curve reached 10–15 attempts earlier. This means that the ultrasound technique, is easier to learn than the nerve stimulation technique although the ultrasound technique is thought to require more highly developed motor and visual skills [18–20]. In our eyes the main reason for this difference is the fact that the staff anaesthesiologist is able to follow the needle track and all needle manipulations of the resident on the screen. Malpositioning and false direction of the needle is better recognized and can be corrected immediately. Integrating visual and tactile information with anatomical knowledge and instructor comments appear to accelerate resident learning. 

The flat part of the learning curve (Figures 1–3) is descriptive for the maximal reached success rate once the skills have been learned (final success rate). The same success rate has been shown in randomised controlled trials comparing ultrasound with nerve stimulator guidance for interscalene [21], infraclavicular [22], and distal sciatic nerve block [23]. For the axillary brachial plexus block our success rate with ultrasound and nerve stimulation is similar to the data of Lo et al. [10]. Nevertheless, the final success rate of 89% after learning ultrasound-guided axillary brachial plexus block is lower than those reported by Chan et al. [9] and Casati et al. [12]. There are three possible explanations for this difference. First of all, in contrast to other studies, we describe the initial learning phase of junior residents acquiring the method for the first time and not terminal success rates or the level at proficiency of experts. Secondly, ultrasound had recently been introduced in our institution prior to the first resident instruction. That means that the teachers learned it only a few months prior to the first resident. Their individual learning curves to perform a block and even more to teach the technique were possibly not at the highest level. This could represent an institutional learning curve bias. However, this is a common situation when a new technique is introduced into clinical practice. Thirdly, the supervision was less rigorous after the first 10 blocks. For the first blocks, the teacher was actively present. After these initial blocks, the staff was on call in the operation theatre. Our generated learning curves show a levelling-off after approximately 15 blocks. Since the knowledge of these results, we recommend a close supervision for at least the first 15 blocks performed by residents.

The most frequent error experienced by novices is to lose visibility of the needle tip as described by Sites et al. [18]. This may contribute to the still high incidence of inadvertent vascular puncture (12.4%) in the axillary region in spite of vessel visualization during ultrasound-guided blocks. Nevertheless, ultrasound guidance dramatically reduced the number of vascular punctures compared to the nerve stimulation technique, as supported by Orebaugh et al. [24]. In other regions, to lose visibility of the needle tip can lead to more severe complications (e.g., pneumothorax with the supraclavicular approach; spinal injection, or damage/injection into the vertebral artery with the interscalene approach). Therefore, we start ultrasound-guided block training with the axillary brachial plexus block first and proceed to other locations only after residents are able to reproducibly and continuously manage to localize and follow the needle tip as supported by Marhofer et al. and Hargett et al. [20, 25]. 

Another advantage of using ultrasound is to improve the patient's comfort by omitting nerve stimulation [12]. Nevertheless, we opted to maintain the nerve stimulator connected, but at a reduced current (0.3–0.5 mA), to help with recognition and avoidance of intraneural needle placement, in cases where the needle tip was poorly visualised. Curiously, Sites et al. [18] and Lo et al. [10] suggest that the use of a nerve stimulator may reduce success rates when used in combination with ultrasound as trainees may prefer the more familiar motor response as an endpoint rather than the ultrasound-visualized perineural spread of local anesthetic. We made the same observation when analyzing our mixed group, residents already familiar with the nerve stimulator showed a smaller upsurge in the learning curve, reaching the same endpoint, but needing more time.

Previous studies have demonstrated that with ultrasound use, the required volume of local anesthetic can be significantly reduced, and this study supports that finding showing a reduction in local anaesthetic volume occurring as early as after the first few nerve blocks [13–16].

**Limitation**

An obvious limitation of this study is the retrospective analysis of anesthesia records. We cannot exclude that every block supplementation by the surgeon was properly recorded. A prospective study would have been of greater significance but is not feasible anymore. It is probable that a staff experienced in ultrasound-guided axillary brachial plexus block would have a better anatomical knowledge and this would bias his teaching of the stimulator technique.

The Monte-Carlo simulation, as a resampling technique was chosen to mimic a statistical population to generate confidence intervals for the curves. Although the Monte-Carlo simulation is a well-accepted method, it is an extrapolation and has, therefore, some limitations as described elsewhere [3]. 

We evaluated the success rates of novices only for the first 20 to 40 blocks. Improvement of this technique may have continued beyond the first 40 blocks due to the constant technique refinement which may have improved the final success rate of experts to a level much higher than our reported 80% for the nerve stimulator and 89% for ultrasound guidance. Even in our institution, the success rates with the nerve stimulator and ultrasound guidance of our advanced learners or expert anesthesiologists is higher and comparable to the success rates reported in the literature.

Obviously learning curves vary between different institutions and learning environments. For example, our learning curves for nerve stimulator-guided blocks are different from learning curves described by Konrad et al. [4]. It was, therefore, important to compare the learning curves for the two different techniques for axillary brachial plexus block under near constant conditions within the same institution, using the same resources, the same teaching staff, and a similar population of residents.

In conclusion, this retrospective analysis of residents trained by two different needle guidance methods suggests that ultrasound permits higher success rates after fewer blocks, especially for residents with no previous training in nerve stimulation. Inadvertent vascular punctures are markedly reduced when using ultrasound guidance, thus, when they do occur they indicate a further need for needle guidance training.

## Figures and Tables

**Table 1:** Preoperative patient characteristics. Data are numbers or mean (±SD).

	Ultrasound	Nerve stimulator	*P*-values
Number of records	259	343	—
Age mean(SD)	47 (±19.65)	46 (±18.16)	.866
Gender (f : m)	99 : 163	127 : 201	.816
Missing Data or not defined	—	15	
BMI	26 (±5.34)	25 (±4.68)	.631
Surgical characteristics			
Bones hand	66 (25.5)	74 (22.2)	
Hand soft tissues	155 (61.0)	187 (56.2)	
Bones forearm	26 (10.0)	37 (16.9)	.058
Forearm soft tissues	12 (4.6)	35 (5.1)	
Missing data or not defined	—	10	
ASA physical status			
I	94 (36.3)	139 (41.9)	
II	122 (47.0)	147 (44.3)	.344
III	43 (16.6)	46 (13.3)	
Missing Data or unclear	—	11	

**Table 2:** Axillary brachial plexus block characteristics. Data are numbers or mean (±SD).

	Ultrasound	Nerve stimulator	*P*-values
Number of records	259	343	—
Block-performing time in minutes	22.1 (±8.4)	34.7 (±12.7)	<.001
Incidence of vessel puncture	32 (12.4)	173 (50.4)	<.001
Volume of local anaesthetics in millilitres	38.5 (±6.3)	46.7 (±6.9)	<.001
Type of local anaesthetics			
Mepivacaine 1%	182 (73.4)	187 (58.3)	
Ropivacaine 0.75%	49 (19.8)	1 (0.3)	
Combination of mepivacaine and bupivacaine	17 (7.9)	133 (41.3)	<.001
Not recorded	11	22	

**Table 3:** Different success rates for both ultrasound and nerve stimulation groups for the cumulated first 10, 20, 30, and 40 attempts with the according confidence intervals. Chi-square test with Bonferroni correction, *P* < .01.

		Number of attempts and success rate (SR) per group				
Attempts			Ultrasound		Stimulator	*P*-value
	Number of residents	*N*	(SR%) (CI ± 95%)	*N*	(SR%) (CI ± 95%)	
10	20	100	(86) (78–92)	100	(68) (58–76)	.002
20	16	179	(88) (82–92)	199	(76) (70–82)	.004
30	13	235	(89) (85–93)	289	(79) (74–83)	.001
40	4	259	(89) (85–92)	343	(80) (75.0–84)	.002
